# High-dose SFRP2 attenuates fibrosis and promotes angiogenesis via Wnt signaling modulation in diabetic erectile dysfunction

**DOI:** 10.1515/biol-2025-1265

**Published:** 2026-01-23

**Authors:** Beom Yong Rho, Min-Ji Choi, Yan Huang, Fitri Rahma Fridayana, Guo Nan Yin, Ji-Kan Ryu

**Affiliations:** National Research Center for Sexual Medicine and Department of Urology, Inha University College of Medicine, Incheon, 22332, Republic of Korea; Program in Biomedical Science & Engineering, Inha University, Incheon, 22332, Republic of Korea

**Keywords:** angiogenesis, erectile dysfunction, fibrosis, SFRP2 protein, Wnt signaling pathway

## Abstract

This study investigated whether high-dose secreted frizzled-related protein 2 (SFRP2) attenuates fibrosis and improves endothelial function under diabetic-like conditions. Diabetic erectile dysfunction (ED) is marked by progressive corpus cavernosum fibrosis and endothelial dysfunction, driven partly by dysregulated Wnt signaling. Primary human corpus cavernosum fibroblasts were stimulated with bone morphogenetic protein 1 (BMP1, 50 ng/mL) or high glucose (HG, 30 mM), with or without SFRP2, and fibrotic responses were assessed by Western blot for collagen I/IV and Wnt markers. Endothelial dysfunction was modeled in human umbilical vein endothelial cells (HUVECs) using tube formation, proliferation, and apoptosis assays. BMP1 and HG increased SFRP2 expression and collagen accumulation, mimicking low-dose SFRP2 effects. In contrast, high-dose SFRP2 (20 µM) suppressed fibrosis, reducing collagen I/IV expression by 40–62 % and downregulating Wnt3a and Wnt5a/b by 40–66 %. High-dose SFRP2 also enhanced angiogenesis and reduced apoptosis by ∼50 % under HG stress. These findings suggest that high-dose SFRP2 mitigates fibrosis and promotes angiogenesis, likely through inhibition of canonical Wnt signaling. As all results were obtained from *in vitro* models, *in vivo* studies are required. Future work will evaluate high-dose SFRP2 in rodent diabetic ED models to determine therapeutic feasibility, safety, and translational potential.

## 1 Introduction

Diabetic erectile dysfunction (ED) is a common complication of diabetes mellitus and is closely associated with fibrosis in penile tissue [1], 2]. Chronic hyperglycemia disrupts vascular homeostasis, leading to fibrosis in the corpus cavernosum and tunica albuginea, which ultimately impairs erectile function [3]. This fibrosis is characterized by collagen deposition, extracellular matrix (ECM) remodeling, and activation of fibroblasts and myofibroblasts, all of which interfere with normal tissue function and vascular dilation [4]. In the diabetic state, these fibrotic changes are amplified by activation of profibrotic signaling pathways, such as transforming growth factor beta (TGF-*β*) and Wnt, which promote excessive ECM production [5]. Importantly, diabetic ED affects approximately 50–75 % of men with long-standing diabetes and is frequently resistant to first-line phosphodiesterase-5 inhibitors, highlighting the urgent clinical need for mechanism-driven therapeutic strategies [1]. However, current treatments primarily target symptomatic vasodilation rather than reversing fibrosis or restoring vascular integrity, limiting their long-term efficacy.

The secreted frizzled-related protein (SFRP) family comprises a group of extracellular glycoproteins that act as key modulators of the Wnt signaling pathway, mainly functioning as negative regulators [6]. Each SFRP contains a cysteine-rich domain structurally homologous to the Frizzled receptor, enabling binding to Wnt ligands and interference with downstream signal transduction [7]. Among these, SFRP2 has emerged as a critical regulator of fibrotic remodeling [8]. Prior studies have demonstrated its biphasic activity: low concentrations enhance BMP1-mediated procollagen processing and promote fibrosis, whereas high concentrations antagonize canonical Wnt signaling and attenuate ECM accumulation [9], [10], [11]. To date, penile-specific evidence for SFRP2 involvement in diabetic ED is extremely limited, and no studies have explored dose-dependent regulation in human corpus cavernosum fibroblasts, representing a significant knowledge gap regarding Wnt-mediated fibrotic and angiogenic balance in diabetic penile tissue.

SFRP2 exhibits dual biological roles relevant to ED pathophysiology. In fibrosis, inhibition of the canonical Wnt pathway may restrain fibroblast-to-myofibroblast transition, reducing excessive collagen deposition. Conversely, SFRP2 has been reported to promote angiogenesis through non-canonical Wnt/Ca^2+^/NFAT signaling and enhance vascular repair [12]. Separating these antifibrotic and angiogenic effects suggests translational potential to simultaneously mitigate fibrosis and restore endothelial function in diabetic ED.

Based on these findings, we hypothesized that high-dose SFRP2 would attenuate fibrosis and enhance angiogenesis in diabetic-like conditions by modulating Wnt signaling in a dose-dependent manner. To test this hypothesis, we evaluated low- and high-dose SFRP2 in primary human corpus cavernosum fibroblasts and human umbilical vein endothelial cells (HUVECs) and assessed fibrosis-related markers, Wnt activation, angiogenesis, and cell survival. Our findings demonstrate dose-dependent functions of SFRP2 and support its potential as a therapeutic target for fibrosis and vascular injury associated with diabetic ED.

## 2 Materials and methods

### 2.1 Ethics statement and study design

All tunica albuginea (TA) tissues used for the isolation of human corpus cavernosum fibroblasts, as well as all experimental animal procedures, were approved by the Institutional Review Board of Inha University (IRB No. 2007-730). Written informed consent was obtained from all human participants prior to tissue collection in accordance with the Declaration of Helsinki. Primary corpus cavernosum fibroblasts were isolated from tunica albuginea tissue obtained from one male donor undergoing penoplasty for congenital penile curvature (age 21). Non-fibrotic regions of the tissue, carefully dissected during surgery, were extracted for subsequent culture.This study aimed to evaluate the role of SFRP2 in mitigating diabetic fibrosis. First, we examined SFRP2 expression in fibroblasts derived from human corpus cavernosum tissue under normal glucose (NG) and high glucose (HG) conditions. Second, under fibrotic conditions induced by BMP1 and HG, we treated the cells with human recombinant SFRP2 and evaluated its antifibrotic effects by assessing changes in ECM molecules and Wnt signaling activity. Finally, we used HUVECs to investigate the effects of SFRP2 on angiogenesis and cell survival. HUVECs were utilized as a surrogate endothelial model due to the limited availability of primary human penile endothelial cells. HUVECs have been shown to recapitulate key physiological features of penile endothelium, including angiogenic behavior, NO-related signaling, and glucose-induced stress responses, supporting their validity in erectile dysfunction–associated vascular research [13]. A schematic overview illustrating the treatment sequence (NG, HG, BMP1, SFRP2) and corresponding analytical endpoints (fibrosis, angiogenesis, apoptosis) has been included to enhance clarity (Supplementary Figure 1).

**Informed consent:** Informed consent has been obtained from all individuals included in this study.

**Ethical approval:** The research related to human use has been complied with all the relevant national regulations, institutional policies and in accordance with the tenets of the Helsinki Declaration, and has been approved by the Institutional Review Board of Inha University (IRB No. 2007-730).

### 2.2 Primary culture and characterization of human corpus cavernosum (CC) fibroblasts

Primary cultures of human corpus cavernosum (CC) fibroblasts were established from TA tissues as previously described [14], 15]. Briefly, tissues were collected in sterile Hank’s balanced salt solution (Gibco, Carlsbad, CA, USA), washed three times with phosphate-buffered saline (PBS), and sectioned into 1–2 mm fragments. Samples were digested in 12.5 mL Dulbecco’s Modified Eagle’s Medium (DMEM; Gibco) containing 0.06 % collagenase A (Sigma-Aldrich, St. Louis, MO, USA) for 1 h at 37 °C in a humidified incubator with 5 % CO_2_. The resulting cell suspension was centrifuged at 400×*g* for 5 min, washed, and plated onto 100-mm culture dishes (Falcon, Becton Dickinson Labware, Franklin Lakes, NJ, USA) in DMEM supplemented with 10 % fetal bovine serum (FBS), 100 U/mL penicillin, and 100 µg/mL streptomycin. The culture medium was replaced every 2 days, and cells at passages 5 to 8 were used for subsequent experiments.

To confirm cell identity, cultured cells were grown on sterile glass coverslips in 12-well plates until nearly confluent and stained with antibodies against CD90 and vimentin (fibroblast markers; R&D Systems Inc., Minneapolis, MN, USA; 1:100), CD31 (endothelial cell marker; Chemicon, Temecula, CA, USA; 1:50), neural/glial antigen 2 (NG2, pericyte marker; Millipore, San Francisco, CA, USA; 1:50), and 4′,6-diamidino-2-phenylindole (DAPI, nuclear marker; Vector Laboratories Inc., Burlingame, CA, USA), as previously described [16]. Fluorescence images were acquired using a K1-Fluo confocal microscope (Nanoscope Systems, Daejeon, Korea).

### 2.3 Cell culture and treatment

HUVECs (Lonza, Cohasset, MN, USA) were cultured according to American Type Culture Collection guidelines, then used between passages 2 and 7. Cells were maintained in M199 medium (Gibco) supplemented with 20 % FBS, 0.5 mg/mL heparin (Sigma-Aldrich), 5 ng/mL recombinant human vascular endothelial growth factor (R&D Systems), and 1 % penicillin/streptomycin on 0.2 % gelatin-coated dishes.

To mimic diabetic conditions and induce fibrosis, serum-starved HUVECs were exposed to either NG (5 mM) or HG (30 mM; Sigma-Aldrich) for 72 h, with or without SFRP2 (20 nM or 20 µM). The SFRP2 concentrations used in this study (20 nM and 20 μM) were selected based on prior reports describing the biphasic activity of SFRP2 on collagen regulation and Wnt signaling, where low doses promote collagen maturation while high doses inhibit canonical Wnt signaling and attenuate fibrosis [9], [10], [11]. Preliminary dose–response experiments confirmed these concentrations as effective ranges in human corpus cavernosum fibroblasts. In additional experiments, fibrosis was induced by pre-treating cells with BMP1 (15, 50, or 100 ng/mL) for 24 h, followed by SFRP2 treatment for 48 h [17]. Vehicle-treated cells (PBS) were used as negative controls for all assays, while BMP1 (50 ng/mL) treatment served as a positive fibrotic control to validate collagen responses. For endothelial assays, NG-treated HUVECs served as baseline physiological controls, and HG treatment represented diabetic-like stress conditions.

### 2.4 Tube formation assay

Endothelial tube formation was evaluated as previously described [18]. Growth factor-reduced Matrigel (50 µL; Becton Dickinson) was added to 96-well plates and allowed to solidify at 37 °C for 10 min. HUVECs pre-treated under NG or HG conditions, with or without SFRP2, were seeded at 5 × 10^4^ cells/well in 150 µL of M199 medium containing 2 % FBS. After 18 h, tube structures were imaged using a CKX41 phase-contrast microscope (Olympus, Tokyo, Japan). The number of master junctions was quantified in a blinded manner from four independent experiments using ImageJ (NIH, v1.34; http://rsbweb.nih.gov/ij/).

### 2.5 Cell migration assay

Cell migration was assessed using the SPL Scar™ Block system (SPL Life Sciences, Pocheon-si, Gyeonggi-do, Korea) to create uniform scratches, as previously described [19]. HUVECs were seeded to >90 % confluence; after 5 h, the blocks were removed. Cells were then incubated in medium containing 2 % FBS and 2 mM thymidine (Sigma-Aldrich) for 24 h. Images were captured with a phase-contrast microscope, and migration was quantified in a blinded manner using ImageJ.

### 2.6 Terminal deoxynucleotidyl transferase-mediated dUTP nick-end labeling (TUNEL) assay

Apoptosis was detected using a TUNEL assay kit (Chemicon, Temecula, CA, USA), according to the manufacturer’s protocol and a previously described method [20]. Digital images of TUNEL-positive cells were acquired using a confocal microscope (K1-Fluo; Nanoscope Systems) at 200 × magnification. Quantification was performed in four independent experiments using ImageJ in a blinded manner.

### 2.7 BrdU incorporation assays

Cell proliferation was evaluated by 5-bromo-2′-deoxyuridine (BrdU) incorporation as previously described [21]. HUVECs were incubated with 10 µM BrdU (Sigma-Aldrich) for 1 h at 37 °C, fixed, and subjected to antigen retrieval. BrdU incorporation was visualized with an anti-BrdU antibody (1:200; Bio-Rad, Hercules, CA, USA) and confocal microscopy. BrdU-positive cells were counted in four randomly selected fields at 200 × magnification using ImageJ in a blinded manner.

### 2.8 Histological examination

For fluorescence histology, cultured cells were fixed with 4 % paraformaldehyde for 15 min at room temperature. Samples were blocked using 1 % bovine serum albumin and incubated overnight at 4 °C with primary antibodies against SFRP2 (Abcam, 1:50), collagen I (Invitrogen, 1:100), and collagen IV (Millipore, 1:100). After samples had been washed with PBS, secondary antibodies were applied: DyLight^®^ 550-conjugated donkey anti-rabbit (Abcam) and Alexa Fluor^®^ 488-conjugated donkey anti-mouse and anti-goat (Jackson ImmunoResearch), all at 1:200 dilution. Samples were mounted in a DAPI-containing solution (Vector Laboratories) for nuclear staining [20]. Images were obtained using a confocal microscope (Nanoscope Systems) with the following laser excitation wavelengths: 405 nm for DAPI, 488 nm for FITC, and 561 nm for TRITC. Detector gain and offset settings were kept constant across experimental groups to enable direct comparison and minimize fluorescence variation. Emission ranges were optimized to avoid spectral overlap. Quantification was performed using ImageJ in a blinded manner across four independent biological replicates.

### 2.9 Western blotting

Protein lysates (50 µg per lane) were separated by sodium dodecyl sulfate–polyacrylamide gel electrophoresis on 4–20 % gradient gels, transferred to polyvinylidene fluoride membranes, and blocked with 5 % nonfat milk. Membranes were probed with primary antibodies against SFRP2 (Abcam, 1:2000), collagen I (Invitrogen, 1:2000), collagen IV (Millipore, 1:2000), Wnt3a and Wnt5a (Cell Signaling Technology, 1:2000), and *β*-actin (Santa Cruz Biotechnology, 1:4000). *β*-actin was used as the internal loading control, and its expression stability under NG and HG conditions was verified by comparing band intensities across representative samples. No significant differences in *β*-actin levels were observed among treatment groups, confirming its suitability for normalization. Detection was performed with an enhanced chemiluminescence system (Amersham Pharmacia Biotech), and band intensities were quantified by densitometry in four independent experiments using ImageJ in a blinded manner.

### 2.10 Statistical analysis

All quantitative data are expressed as means ± standard errors of the mean from at least four independent experiments. Normality of data distribution was assessed using the Shapiro–Wilk test, and homogeneity of variances was evaluated using Levene’s test prior to applying parametric statistical tests. Unpaired Student’s *t*-tests were used for comparisons between two groups, and one-way ANOVA followed by Tukey’s post hoc test was applied for multiple group comparisons. Statistical analyses were carried out using GraphPad Prism 8 (GraphPad Software Inc., San Diego, CA, USA). A P-value < 0.05 was considered statistically significant.

## 3 Results

### 3.1 Primary culture and identification of human CC fibroblasts

**Figure 1:**
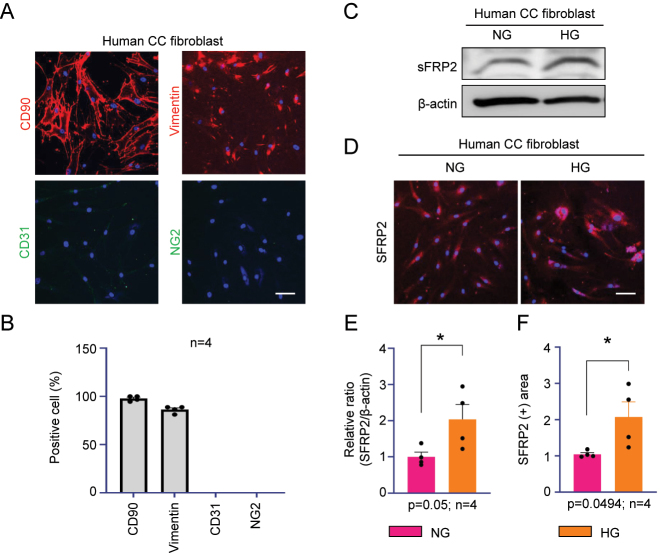
SFRP2 expression is increased in human CC fibroblasts under HG conditions. (A) Characterization of human CC fibroblasts by immunofluorescence staining showing positive expression of fibroblast markers CD90 and vimentin, and absence of the endothelial marker CD31 and the pericyte marker NG2. (B) Quantification of the cell marker-positive percentage in immunofluorescence images. (C) Representative western blots for SFRP2 under NG (5 mM) and HG (30 mM) conditions. (D) SFRP2 immunostaining (red) in human CC fibroblasts under NG and HG conditions. Nuclei were labeled with the DNA dye DAPI (blue). (E) Quantification of the SFRP2/β-actin ratio by densitometry. (F) Quantification of the SFRP2-positive (+) area in immunofluorescence images. Data are presented as mean ± SEM (*n* = 4). The relative ratio in the NG was set to 1. *P < 0.05. Scale bars, 100 µm. CC, corpus cavernosum; NG, normal glucose; HG, high glucose.

To investigate the effects of SFRP2 on fibrosis in human CC fibroblasts under HG conditions, primary fibroblasts were successfully isolated from human CC TA tissue. Primary human corpus cavernosum fibroblasts displayed positive immunofluorescence staining for CD90 and vimentin and were negative for CD31 and NG2, confirming fibroblast identity (Figure 1A). Quantification of fluorescence-positive cells revealed markedly higher expression of CD90 and vimentin compared with CD31 and NG2 (Figure 1B). All experiments were performed using cells at passages 3–8. These findings, consistent with a previous report [14], confirmed that the isolated cells were human CC fibroblasts suitable for subsequent analyses.

### 3.2 SFRP2 expression increases in human CC fibroblasts under HG conditions

To examine the effect of HG on SFRP2 expression, primary CC fibroblasts were cultured under NG and HG conditions. Exposure to HG (30 mM) for 48 h significantly increased SFRP2 expression compared with NG conditions, as shown by densitometric analysis of Western blots (Figure 1C and 1E) and immunofluorescence staining (Figure 1D and 1F). Quantification demonstrated a ∼2.1-fold elevation in SFRP2 protein levels relative to NG (p = 0.0494). These results indicate that a hyperglycemic, diabetes-like environment can upregulate SFRP2 expression in penile fibroblasts and may initiate early fibrotic signaling.

### 3.3 BMP1- and HG-induced SFRP2 expression corresponds to the functional range of low-dose SFRP2

**Figure 2:**
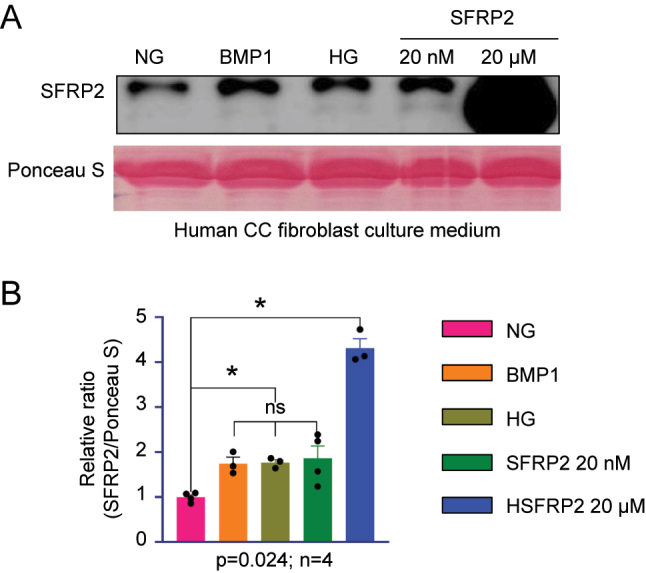
BMP1 and HG induce SFRP2 expression at levels comparable to low-dose SFRP2. (A) Representative western blots of culture medium showing secreted SFRP2 levels in human CC fibroblasts treated with BMP1 (50 ng/mL), HG (30 mM), low-dose SFRP2 (20 nM), or high-dose SFRP2 (20 μM). Ponceau S staining served as a loading control. (B) Quantification of the SFRP2/Ponceau S ratio by densitometry. Data are presented as mean ± SEM (*n* = 3–4). The relative ratio in the NG was set to 1.*P < 0.05, ***P < 0.001; ns, not significant. CC, corpus cavernosum; HG, high glucose.

The profibrotic effects of SFRP2 are dose-dependent, such that low and high doses produce distinct biological outcomes [11]. To determine whether the HG-induced upregulation of SFRP2 corresponds to low or high functional doses, endogenous SFRP2 levels were compared in fibroblasts treated with BMP1 (a positive control for fibrosis induction), HG, low-dose SFRP2 (20 nM), and high-dose SFRP2 (20 µM). Western blot analysis showed that BMP1- and HG-induced SFRP2 expression was comparable to that in the low-dose SFRP2 group (Figure 2). These results suggest that BMP1- and HG-induced SFRP2 upregulation falls within the low-dose functional range (20 nM) and mimics the profibrotic effects observed under these stimuli, indicating that both treatments functionally recapitulate a low-dose SFRP2 response. Interpretation of low-dose functional activity was based on relative densitometric quantification of Western blot band intensity normalized to *β*-actin rather than absolute protein levels.

### 3.4 High-dose SFRP2 attenuates BMP1-induced collagen expression and Wnt signaling activation in human CC fibroblasts

**Figure 3:**
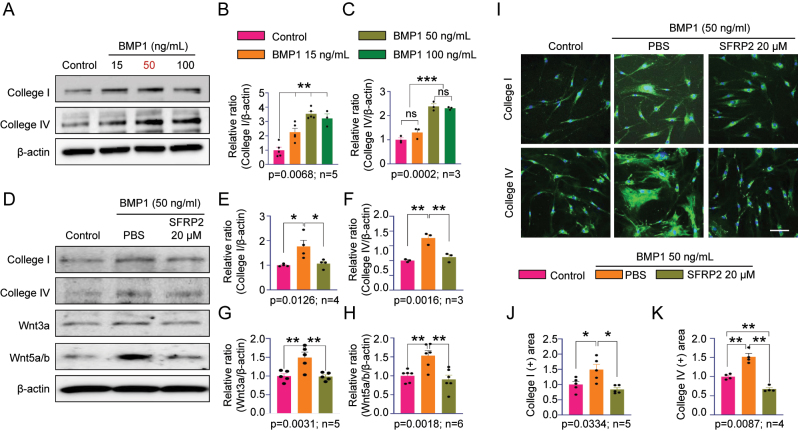
High-dose SFRP2 inhibits BMP1-induced collagen expression and Wnt signaling in human CC fibroblasts. (A) Representative western blot showing dose-dependent induction of collagen I and collagen IV by BMP1 (15, 50, and 100 ng/mL). (B, C) Quantification of collagen I and collagen IV relative to *β*-actin. (D) Representative western blot showing that co-treatment with SFRP2 (20 μM) suppresses BMP1-induced expression of collagen I, collagen IV, Wnt3a, and Wnt5a/b. (E–H) Quantitative analysis of collagen and Wnt markers. (I) Immunofluorescence images of collagen I and collagen IV (green) in human CC fibroblasts under BMP1 ± SFRP2 treatment. (J, K) Quantification of collagen-positive areas. Data are presented as mean ± SEM (*n* = 3–5). The relative ratio in the Control was set to 1. *P < 0.05, **P < 0.01, ***P < 0.001; ns, not significant. Scale bars, 100 µm. CC, corpus cavernosum.

High doses of SFRP2 are known to inhibit the canonical Wnt signaling pathway and suppress collagen-processing enzyme activity, thereby mitigating fibrotic progression [11]. Additionally, SFRP2 exerts pro-angiogenic and cardioprotective effects via modulation of both canonical and non-canonical Wnt signaling pathways [22]. To assess the antifibrotic effect of high-dose SFRP2, human CC fibroblasts were stimulated with BMP1 at various concentrations (15, 50, and 100 ng/mL). As expected, western blot analysis revealed dose-dependent increases in collagen I and collagen IV expression, with maximal induction at 50–100 ng/mL (Figure 3A–C). Based on these results, 50 ng/mL BMP1 was selected for subsequent co-treatment with recombinant SFRP2 (20 µM). High-dose SFRP2 substantially reduced the BMP1-induced expression of both collagen I and collagen IV (Figure 3D–F), with statistically significant differences when compared with BMP1 alone (p < 0.01). Furthermore, SFRP2 suppressed BMP1-induced activation of the Wnt signaling pathway, as indicated by decreased protein levels of Wnt3a and Wnt5a/b (Figure 3D, 3G, 3H). Quantitative densitometry confirmed reductions of approximately 40–66 % relative to BMP1 treatment alone (p < 0.01). Immunofluorescence analysis supported these findings, revealing increased deposition of collagen I and collagen IV in the BMP1 + PBS group, whereas co-treatment with SFRP2 substantially reduced collagen accumulation, in some cases below control levels (Figure 3I–K). These results demonstrate that high-dose SFRP2 effectively inhibits BMP1-induced fibrosis by downregulating collagen production and suppressing canonical Wnt signaling activity in human CC fibroblasts.

### 3.5 High-dose SFRP2 inhibits HG-induced fibrosis via the Wnt pathway

**Figure 4:**
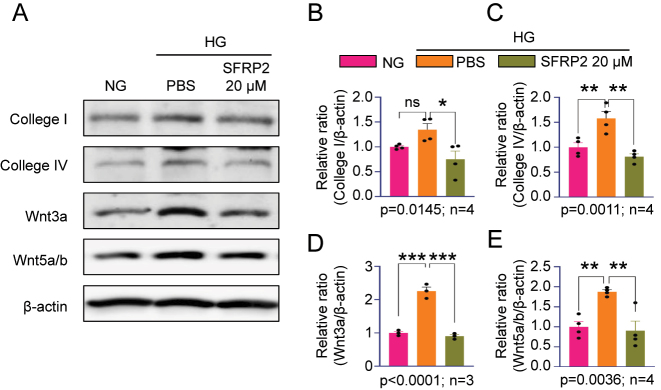
High-dose SFRP2 suppresses HG-induced collagen expression and Wnt signaling in human CC fibroblasts. (A) Representative western blot showing collagen I, collagen IV, and Wnt signaling proteins (Wnt3a and Wnt5a/b) in human CC fibroblasts cultured under NG, HG with PBS, or HG with high-dose SFRP2 (20 μM). (B–E) Quantitative analysis of protein expression relative to *β*-actin, including collagen I (B), collagen IV (C), Wnt3a (D), and Wnt5a/b (E). Data are presented as mean ± SEM (*n* = 3–4). The relative ratio in the NG was set to 1. *P < 0.05, **P < 0.01, ***P < 0.001; ns, not significant. CC, corpus cavernosum; NG, normal glucose; HG, high glucose; PBS, phosphate-buffered saline.

Chronic exposure to HG is a well-recognized driver of fibrotic remodeling in multiple tissues, primarily through activation of fibroblasts and their differentiation into myofibroblasts, which increases ECM protein synthesis and collagen deposition via profibrotic signaling pathways such as TGF-*β* and Wnt [23]. In this study, human CC fibroblasts were exposed to HG (30 mM) for 48 h prior to SFRP2 treatment to maintain consistency with earlier experimental conditions. Accordingly, human CC fibroblasts were cultured under HG (30 mM) conditions with or without recombinant SFRP2 (20 µM). Western blot analysis showed that HG significantly increased collagen I and collagen IV expression compared with NG controls (Figure 4A–C). Co-treatment with SFRP2 considerably reduced HG-induced collagen expression to near or below baseline levels. HG exposure also increased activation of canonical Wnt signaling, as indicated by upregulation of Wnt3a and Wnt5a/b. High-dose SFRP2 treatment effectively downregulated these Wnt ligands, indicating suppression of Wnt signaling activity (Figure 4A, 4D, 4E). Our findings suggest that high-dose SFRP2 mitigates HG-induced fibrotic remodeling by inhibiting Wnt signaling activation and reducing ECM protein overproduction in penile fibroblasts.

### 3.6 High-dose SFRP2 promotes angiogenesis and enhances endothelial cell survival under HG conditions

**Figure 5:**
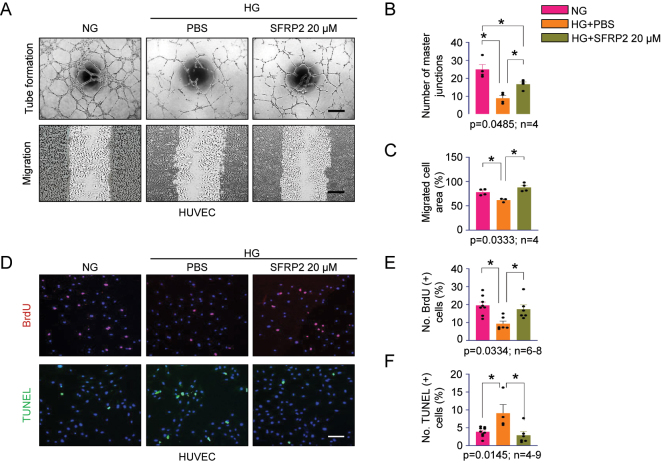
High-dose SFRP2 promotes angiogenesis, migration, and survival of endothelial cells under HG conditions. (A) Representative images from tube formation and migration assays in human umbilical vein endothelial cells (HUVECs) cultured under NG, HG with PBS, or HG with high-dose SFRP2 (20 μM). (B) Quantification of tube formation based on the number of master junctions. (C) Quantification of endothelial cell migration by measuring the migrated area. (D) Representative immunofluorescence images showing BrdU-positive (red) proliferating cells and TUNEL-positive (green) apoptotic cells, with nuclei stained by DAPI (blue). (E) Quantification of BrdU-positive proliferating cells. (F) Quantification of TUNEL-positive apoptotic cells. Data are presented as mean ± SEM (*n* = 3–9). *P < 0.05, **P < 0.01, ***P < 0.001. Scale bars, 100 µm. NG, normal glucose; HG, high glucose; PBS, phosphate-buffered saline; BrdU, 5-bromo-2′-deoxyuridine; TUNEL, terminal deoxynucleotidyl transferase dUTP nick-end labeling.

Chronic HG exposure is known to impair angiogenesis and induce apoptosis, thereby compromising endothelial function and contributing to microvascular dysfunction in diabetic complications such as retinopathy, nephropathy, and ED [20]. To assess the pro-angiogenic and cytoprotective potential of high-dose SFRP2 under diabetic-like conditions, HUVECs were treated with HG (30 mM) in the presence or absence of recombinant SFRP2 (20 µM). Tube formation and migration assays demonstrated that SFRP2 significantly enhanced angiogenic and migratory activity compared with the PBS-treated group under HG conditions (Figure 5A–C). To further evaluate whether these beneficial effects were independent of off-target or cytotoxic responses, BrdU incorporation and TUNEL assays were performed to assess cell proliferation and apoptosis under the same treatment conditions. BrdU staining revealed increased endothelial cell proliferation in the high-dose SFRP2 group, whereas TUNEL staining demonstrated reduced apoptosis (Figure 5D–F). The absence of increased apoptosis or impaired cell proliferation indicates that high-dose SFRP2 does not exert detectable cytotoxic effects, supporting the safety of the treatment concentration used. Collectively, these results indicate that high-dose SFRP2 improves endothelial cell function by promoting angiogenesis, increasing proliferation, and reducing apoptosis under hyperglycemic conditions, suggesting its potential to restore vascular integrity in diabetic complications.

## 4 Discussion

Diabetic ED is a multifactorial complication arising from metabolic, vascular, and fibrotic abnormalities; tissue fibrosis and vascular dysfunction play central roles in its pathogenesis [1]. Chronic hyperglycemia promotes ECM remodeling, myofibroblast activation, and vascular injury primarily through activation of profibrotic signaling pathways, particularly TGF-*β* and Wnt [24]. Despite advances in mechanistic understanding, therapies that directly target these fibrotic and vascular components remain limited. In this study, we used *in vitro* models of BMP1- and HG-induced fibrosis and endothelial dysfunction to investigate the functional role of SFRP2, a Wnt pathway regulator with dose-dependent effects on fibrosis and vascular biology [11]. BMP1 and HG converge on Wnt activation and ECM production via distinct upstream mechanisms – BMP1 enhances procollagen processing and TGF-*β* signaling [25], 26], whereas HG induces oxidative stress and activates PKC, NF-κB, and TGF-*β*1, leading to inhibition of glycogen synthase kinase 3 beta (GSK3*β*) and disruption of non-canonical Wnt/Ca^2+^/NFAT signaling in endothelial cells [27]. Within this context, our data demonstrate that high-dose SFRP2 can counteract these profibrotic and pro-apoptotic cues in diabetic-like settings.

First, we found that SFRP2 expression was modestly upregulated in CC fibroblasts exposed to BMP1 or HG, and that these levels were comparable to those induced by exogenous low-dose SFRP2 (20 nM), suggesting that diabetic-like stimuli elevate SFRP2 into a biologically active, low-dose range. Consistent with prior reports that low SFRP2 can paradoxically enhance fibrosis via BMP1-mediated collagen processing [11], 28], both BMP1 and HG increased collagen I and IV expression, mimicking low-dose SFRP2. In contrast, high-dose SFRP2 (20 µM) markedly reduced BMP1- and HG-induced collagen I/IV expression and downregulated Wnt3a and Wnt5a/b, consistent with inhibition of canonical Wnt signaling. Mechanistically, suppression of these ligands would be expected to limit *β*-catenin stabilization and restore GSK3β activity, thereby attenuating transcription of profibrotic genes such as COL1A1 and COL4A1 [29], although this interpretation remains presumptive and requires direct validation. To further dissect SFRP2-mediated modulation of fibroblast phenotype, future studies will assess myofibroblast-associated markers such as *α*-SMA and fibronectin, as well as perform qPCR and immunoprecipitation analyses to distinguish transcriptional from ligand-sequestration mechanisms. The observation that high-dose SFRP2 also reduced collagen I/IV and Wnt3a/Wnt5a/b in BMP1-stimulated fibroblasts in the absence of hyperglycemia supports a predominantly direct effect on Wnt/BMP1–ECM signaling, although potential contributions from SFRP2-induced metabolic changes under HG (e.g., oxidative or mitochondrial stress) cannot be excluded.

Beyond its antifibrotic effects in fibroblasts, SFRP2 exerted a robust pro-angiogenic and cytoprotective influence on endothelial cells under hyperglycemic conditions [20]. In HUVECs exposed to HG, high-dose SFRP2 enhanced tube formation and migration, increased proliferation (BrdU incorporation), and reduced apoptosis (TUNEL staining), indicating improved endothelial survival and angiogenic capacity. These findings are consistent with a role for SFRP2 in engaging non-canonical Wnt/Ca^2+^/NFAT/Fzd5 signaling previously implicated in cardiac and vascular repair [22]. Future work will determine whether these effects are VEGF-dependent or predominantly mediated through Ca^2+^/NFAT signaling by assessing VEGF expression, NFAT nuclear translocation, CaMKII activation, and intracellular Ca^2+^ flux in HUVECs. Given that our experiments used HUVECs and fibroblasts from a single non-diabetic donor, *in vivo* and ex vivo validation – including Matrigel plug assays and fibroblast–endothelial co-culture models under diabetic conditions – will be essential to confirm the translational relevance of SFRP2-mediated endothelial protection and angiogenesis in the penile microenvironment.

Taken together, our results demonstrate that SFRP2 acts in a dual, dose-dependent manner: at low levels, it may sustain or enhance fibrotic signaling, whereas at high levels, it exhibits antifibrotic and vasoprotective effects. By reducing collagen deposition and restoring endothelial viability and angiogenesis, high-dose SFRP2 directly targets the two core determinants of erectile failure in diabetes – corpus cavernosum stiffness and impaired NO-mediated vasodilation. These coordinated molecular effects are expected to preserve cavernosal compliance and vascular responsiveness, supporting improved erectile function *in vivo*. These findings have important implications not only for diabetic ED but also for other fibrosis-associated vascular disorders. They also suggest that modulation of SFRP2 could serve as a promising therapeutic approach to reverse fibrosis and restore endothelial function in diabetic penile tissue. The rationale for the two selected doses of SFRP2 (20 nM and 20 µM) was based on both literature evidence and preliminary experiments [11], 28], 30]. Consistent with this, our pilot dose-response experiments in fibroblasts indicated that SFRP2 (20 µM) was necessary to significantly reduce collagen I/IV expression under BMP1- or HG-induced conditions, while nanomolar doses produced minimal inhibitory activity. Thus, the 1,000-fold difference between the two doses (20 nM vs 20 µM) was intentionally exploratory, designed to qualitatively capture the biphasic switch of SFRP2 activity rather than a strict pharmacologic gradient.

SFRP2 also exhibits functional properties that distinguish it from other secreted frizzled-related proteins and broadly acting antifibrotic agents [11], 28]. Whereas SFRP1, SFRP4, DKK proteins, and small-molecule Wnt or TGF-*β* inhibitors typically exert unidirectional antifibrotic effects in renal, cardiac, or dermal models without a clear dose-dependent switch, SFRP2 uniquely enhances BMP1-mediated procollagen processing at low concentrations while antagonizing canonical Wnt signaling and promoting angiogenesis at higher concentrations [31]. Our findings in primary human corpus cavernosum fibroblasts and HUVECs align with this bifunctional profile: BMP1- and HG-induced endogenous SFRP2 corresponds to a low-dose, profibrotic range, whereas exogenous high-dose SFRP2 suppresses Wnt3a/Wnt5a/b, reduces collagen accumulation, and improves endothelial survival and tube formation under hyperglycemic conditions. This dual antifibrotic and pro-angiogenic behavior highlights SFRP2 as a uniquely tunable Wnt modulator within diabetic erectile tissue and offers advantages over non-selective Wnt blockade used in other diabetic vascular or fibrotic complications.

From a translational perspective, our findings imply the presence of a therapeutic window in which SFRP2 concentrations must exceed endogenous low-dose, profibrotic levels yet remain below thresholds that may induce off-target effects. The lower boundary is approximated by BMP1-/HG-induced endogenous SFRP2 expression, whereas the upper boundary likely lies within the micromolar range, where antifibrotic and pro-angiogenic effects plateau without cytotoxicity. Achieving and maintaining such concentrations *in vivo* will likely require localized intracavernosal administration. Injectable hydrogels, microparticles, and nanoparticle-based systems – previously shown to support controlled release and erectile recovery in preclinical ED models – represent feasible delivery platforms for SFRP2. Future work will explore intermediate dosing (0.1–5 µM) and characterize pharmacokinetic/pharmacodynamic profiles to establish a clinically relevant therapeutic window.

Clinically, SFRP2-based therapy is best positioned as a disease-modifying adjunct rather than a replacement for current treatments. PDE5 inhibitors enhance NO–cGMP signaling but do not reverse fibrosis or endothelial loss, leading to frequent therapeutic failure in long-standing diabetes [1]. By restoring cavernosal structure, SFRP2 may improve responsiveness to PDE5 inhibitors and other vasodilatory agents. Because systemic Wnt modulation raises concerns regarding tissue homeostasis, bone remodeling, and tumorigenesis, localized delivery and controlled-release formulations should remain the main focus of translational development, accompanied by thorough preclinical safety evaluation.

A key limitation of this study is the exclusive use of *in vitro* systems derived from a single non-diabetic donor, which cannot fully represent the inflammatory, metabolic, and neurovascular complexity of diabetic penile tissue. Mechanistic inference was limited by the absence of pathway inhibitor or genetic knockdown validation, as well as lack of downstream readouts such as *β*-catenin or TCF/LEF activity. Additionally, the *in vitro* 20 µM concentration requires *in vivo* assessment of safety, pharmacologic feasibility, and long-term tissue effects. Future studies will incorporate multiple donor-derived lines, expanded dose–response analysis, and diabetic animal models (STZ-induced, db/db, Akita, and cavernous nerve injury) to allow integrated evaluation of erectile hemodynamics, vascular regeneration, fibrosis, and Wnt pathway modulation.

## 5 Conclusions

In summary, SFRP2 demonstrates a dual dose-dependent regulatory mechanism: at low concentrations it supports profibrotic signaling through BMP1-enhanced collagen processing, whereas at higher concentrations it inhibits canonical Wnt signaling, suppresses extracellular matrix accumulation, and promotes angiogenesis. These findings highlight SFRP2 as a promising therapeutic candidate for diabetic erectile dysfunction and other fibrosis-associated vascular disorders. Translational advancement will require *in vivo* pharmacodynamic validation, testing of intermediate dosing ranges to establish a therapeutic window, and the development of localized delivery strategies such as biomaterial-based or nanoparticle-assisted administration. Together, these steps define a clear roadmap toward clinical feasibility.

## Supplementary Material

Supplementary Material

## References

[1] Defeudis G, Mazzilli R, Tenuta M, Rossini G, Zamponi V, Olana S (2022). Erectile dysfunction and diabetes: a melting pot of circumstances and treatments. Diabetes Metab Res Rev.

[2] Xia M, Yuan Y, Fang D, Tan X, Zhao F, Li X (2025). Blocking TSP1 ameliorates diabetes mellitus-induced erectile dysfunction by inhibiting the TGF-beta/SMAD pathway. World J Mens Health.

[3] Giri B, Dey S, Das T, Sarkar M, Banerjee J, Dash SK (2018). Chronic hyperglycemia mediated physiological alteration and metabolic distortion leads to organ dysfunction, infection, cancer progression and other pathophysiological consequences: an update on glucose toxicity. Biomed Pharmacother.

[4] Wang K, Wen D, Xu X, Zhao R, Jiang F, Yuan S (2023). Extracellular matrix stiffness-the central cue for skin fibrosis. Front Mol Biosci.

[5] Yousefi F, Shabaninejad Z, Vakili S, Derakhshan M, Movahedpour A, Dabiri H (2020). TGF-beta and WNT signaling pathways in cardiac fibrosis: non-coding RNAs come into focus. Cell Commun Signal.

[6] Liu LB, Chen XD, Zhou XY, Zhu Q (2018). The Wnt antagonist and secreted frizzled-related protein 5: implications on lipid metabolism, inflammation, and type 2 diabetes mellitus. Biosci Rep.

[7] Guan H, Zhang J, Luan J, Xu H, Huang Z, Yu Q (2021). Secreted frizzled related proteins in cardiovascular and metabolic diseases. Front Endocrinol.

[8] Lin H, Angeli M, Chung KJ, Ejimadu C, Rosa AR, Lee T (2016). sFRP2 activates Wnt/beta-catenin signaling in cardiac fibroblasts: differential roles in cell growth, energy metabolism, and extracellular matrix remodeling. Am J Physiol Cell Physiol.

[9] Napoli M, Bauer J, Bonod C, Vadon-Le Goff S, Moali C (2024). PCPE-2 (procollagen C-proteinase enhancer-2): the non-identical twin of PCPE-1. Matrix Biol.

[10] Somanader DVN, Zhao P, Widdop RE, Samuel CS (2024). The involvement of the Wnt/beta-catenin signaling cascade in fibrosis progression and its therapeutic targeting by relaxin. Biochem Pharmacol.

[11] Wu Y, Liu X, Zheng H, Zhu H, Mai W, Huang X (2020). Multiple roles of sFRP2 in cardiac development and cardiovascular disease. Int J Biol Sci.

[12] Courtwright A, Siamakpour-Reihani S, Arbiser JL, Banet N, Hilliard E, Fried L (2009). Secreted frizzle-related protein 2 stimulates angiogenesis via a calcineurin/NFAT signaling pathway. Cancer Res.

[13] Kwon MH, Ryu JK, Kim WJ, Jin HR, Song KM, Kwon KD (2013). Effect of intracavernous administration of angiopoietin-4 on erectile function in the streptozotocin-induced diabetic mouse. J Sex Med.

[14] Piao S, Choi MJ, Tumurbaatar M, Kim WJ, Jin HR, Shin SH (2010). Transforming growth factor (TGF)-beta type I receptor kinase (ALK5) inhibitor alleviates profibrotic TGF-beta1 responses in fibroblasts derived from Peyronie’s plaque. J Sex Med.

[15] Ryu JK, Kim WJ, Choi MJ, Park JM, Song KM, Kwon MH (2013). Inhibition of histone deacetylase 2 mitigates profibrotic TGF-beta1 responses in fibroblasts derived from Peyronie’s plaque. Asian J Androl.

[16] Yin GN, Ryu JK, Kwon MH, Shin SH, Jin HR, Song KM (2012). Matrigel-based sprouting endothelial cell culture system from mouse corpus cavernosum is potentially useful for the study of endothelial and erectile dysfunction related to high-glucose exposure. J Sex Med.

[17] Detaille D, Guigas B, Chauvin C, Batandier C, Fontaine E, Wiernsperger N (2005). Metformin prevents high-glucose-induced endothelial cell death through a mitochondrial permeability transition-dependent process. Diabetes.

[18] Yang J, Yin GN, Kim DK, Han AR, Lee DS, Min KW (2023). Crystal structure of LRG1 and the functional significance of LRG1 glycan for LPHN2 activation. Exp Mol Med.

[19] Ock J, Wu J, Liu FY, Fridayana FR, Niloofar L, Vo MN (2023). Heme-binding protein 1 delivered via pericyte-derived extracellular vesicles improves neurovascular regeneration in a mouse model of cavernous nerve injury. Int J Biol Sci.

[20] Liu FY, Cho YL, Fridayana FR, Niloofar L, Vo MN, Huang Y (2025). MT-100, a human Tie2-agonistic antibody, improves penile neurovasculature in diabetic mice via the novel target Srpx2. Exp Mol Med.

[21] Ock J, Suh JK, Hong SS, Kang JH, Yin GN, Ryu JK (2023). IGFBP5 antisense and short hairpin RNA (shRNA) constructs improve erectile function by inducing cavernosum angiogenesis in diabetic mice. Andrology.

[22] Akoumianakis I, Polkinghorne M, Antoniades C (2022). Non-canonical WNT signalling in cardiovascular disease: mechanisms and therapeutic implications. Nat Rev Cardiol.

[23] Gyorfi AH, Matei AE, Distler JHW (2018). Targeting TGF-beta signaling for the treatment of fibrosis. Matrix Biol.

[24] Rask-Madsen C, King GL (2013). Vascular complications of diabetes: mechanisms of injury and protective factors. Cell Metab.

[25] Zent J, Guo LW (2018). Signaling mechanisms of myofibroblastic activation: outside-in and inside-out. Cell Physiol Biochem.

[26] Zhang Y, Jin D, Kang X, Zhou R, Sun Y, Lian F (2021). Signaling pathways involved in diabetic renal fibrosis. Front Cell Dev Biol.

[27] Deng Z, Fan T, Xiao C, Tian H, Zheng Y, Li C (2024). TGF-beta signaling in health, disease, and therapeutics. Signal Transduct Target Ther.

[28] Kobayashi K, Luo M, Zhang Y, Wilkes DC, Ge G, Grieskamp T (2009). Secreted Frizzled-related protein 2 is a procollagen C proteinase enhancer with a role in fibrosis associated with myocardial infarction. Nat Cell Biol.

[29] von Marschall Z, Fisher LW (2010). Secreted Frizzled-related protein-2 (sFRP2) augments canonical Wnt3a-induced signaling. Biochem Biophys Res Commun.

[30] Mastri M, Shah Z, Hsieh K, Wang X, Wooldridge B, Martin S (2014). Secreted Frizzled-related protein 2 as a target in antifibrotic therapeutic intervention. Am J Physiol Cell Physiol.

[31] Hsueh YC, Hodgkinson CP, Gomez JA (2021). The role of Sfrp and DKK proteins in cardiomyocyte development. Physiol Rep.

