# Supplementary figures and images for: High-dose SFRP2 attenuates fibrosis and promotes angiogenesis via Wnt signaling modulation in diabetic erectile dysfunction
Source: Open Life Sci. 2026 Jan 23;21(1):20251265. doi: 10.1515/biol-2025-1265 (PMC12917599; doi:10.1515/biol-2025-1265)

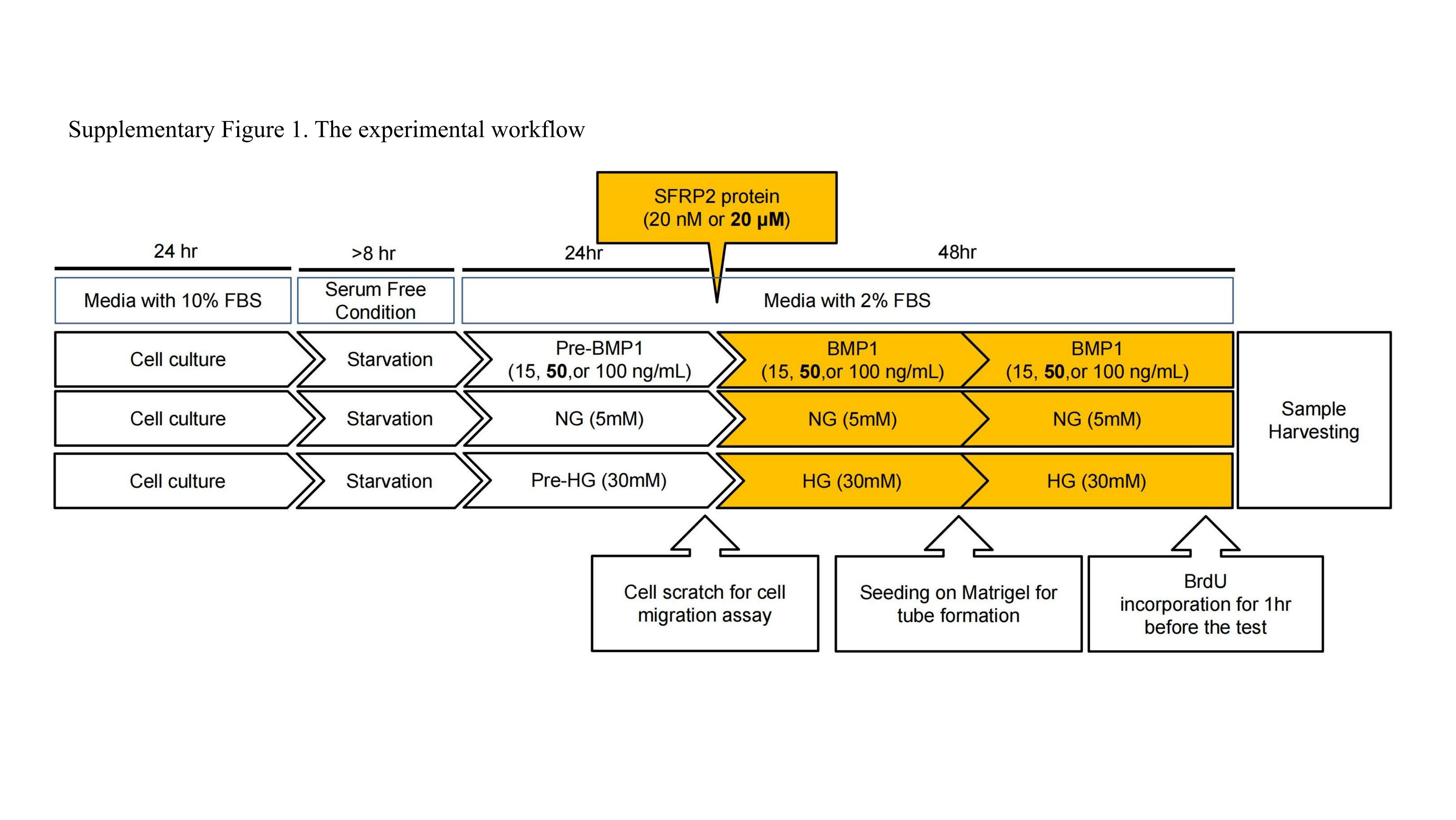

Supplement: Supplementary file 1 — Supplementary Material [file j_biol-2025-1265_suppl_001.jpg]
